# Considerations on the Identity and Diversity of Organisms Affiliated with *Sphingobacterium multivorum*—Proposal for a New Species, *Sphingobacterium paramultivorum*

**DOI:** 10.3390/microorganisms9102057

**Published:** 2021-09-29

**Authors:** Yanfang Wang, Jolanda K. Brons, Jan Dirk van Elsas

**Affiliations:** Cluster of Microbial Ecology, Groningen Institute for Evolutionary Life Sciences, University of Groningen, 9747 AG Groningen, The Netherlands; j.k.brons@rug.nl

**Keywords:** average nucleotide identity, *Sphingobacterium paramultivorum*, *Sphingobacterium multivorum*, species cluster

## Abstract

Plant biomass offers great potential as a sustainable resource, and microbial consortia are primordial in its bioconversion. The wheat-straw-biodegradative bacterial strain w15 has drawn much attention as a result of its biodegradative potential and superior degradation performance in bacterial-fungal consortia. Strain w15 was originally assigned to the species *Sphingobacterium multivorum* based on its 16S ribosomal RNA (rRNA) gene sequence. A closer examination of this taxonomic placement revealed that the sequence used has 98.9% identity with the ‘divergent’ 16S rRNA gene sequence of *S. multivorum* NCTC 11343^T^, yet lower relatedness with the canonical 16S rRNA sequence. A specific region of the gene, located between positions 186 and 210, was found to be highly variable and determinative for the divergence. To solve the identity of strain w15, genome metrics and analyses of ecophysiological niches were undertaken on a selection of strains assigned to *S. multivorum* and related species. These analyses separated all strains into three clusters, with strain w15, together with strain BIGb0170, constituting a separate radiation, next to *S. multivorum* and *S. siyangense*. Moreover, the strains denoted FDAARGOS 1141 and 1142 were placed inside *S. siyangense*. We propose the renaming of strains w15 and BIGb0170 as members of the novel species, coined *Sphingobacterium paramultivorum*.

## 1. Introduction

In our studies on bacterial-fungal consortia that are able to degrade, and thrive on, wheat straw (WS), consistent evidence was obtained for the tenet that organisms identified as *Sphingobacterium multivorum* are consistently present and have defined roles in WS degradation processes [1,2]. A key facet of these *Sphingobacterium*-like organisms is their potential to produce a broad range of carbohydrate-active proteins, of which particular endo-1,4-beta-xylanases, alpha-l-arabinofuranosidases and alpha-l-fucosidases (belonging to CAZy families GH3, GH10, GH43, GH51, GH67 and GH95) stand out as having likely roles in consortial degradation processes [2]. In the course of our studies, a strain (denoted w15), preliminarily identified as *S. multivorum* based on its 16S rRNA sequence, was selected for further work, as it revealed a remarkable behavior when grown in combination with the fungus *Coniochaeta ligniaria* and the bacterium *Citrobacter freundii* so4 [3]. Other studies have also pointed to a role of *S. multivorum* (and alike) strains in the degradation of organic polymers from plant remains [4,5], keratan (endo-/β-galactosidase activity) [6], arabinoxylan [7], hexaconazole triazole fungicide [8] as well as petroleum and polycyclic aromatic hydrocarbon [9,10,11]. Moreover, a role in drought tolerance and growth promotion in tomato has been suggested [12]. On the other hand, *S. multivorum* strains have been isolated from clinical specimens, often as organisms involved in diverse kinds of opportunistic infections [13], causing bacteremia and acute meningitis in young immunocompetent adults [14]; and being involved in multiple myeloma [15] and cystic fibrosis [16].

This ecological versatility of *S. multivorum* strains is another clear motif to further investigate the identity of strain w15. Although its full genome sequence has been produced [17], it has not been fully explored, and hence an examination of its characteristics regarding identity was deemed necessary. We thus examined the available data regarding the origin, ecological niche, nature and diversity of, next to strain w15, selected *S. multivorum* and related strains, including two type strains present in different strain collections (NCTC 11343^T^, DSM 11691^T^).

The bacterial species *S. multivorum* derives from an early analysis of a group of 28 strains that originally fell within the genus *Flavobacterium* as a well-defined cluster (group IIk, biotype 2) [13]. Based on their coherent characteristics, these 28 strains were classified as forming one taxonomically concise, new species, coined *Flavobacterium multivorum*. The type strain, deposited in the NCTC culture collection, is the aforementioned NCTC 11343^T^. Briefly, the species, then considered to be typically clinical, was described as an aerobic, Gram-negative, nonmotile and oxidative bacterium, forming rod-shaped cells and growing at mesophilic temperatures. It was consistently able to produce acid from a range of carbohydrates. Strikingly, considerable resistance to a broad range of antimicrobial agents was found across the strains [13]. Later, based on the finding of high levels of sphingophospholipids as cellular lipid components, Yabuuchi et al. [18] founded the novel genus *Sphingobacterium* and transferred *F. multivorum* to it, yielding *Sphingobacterium multivorum* [13,18]. Currently, the genus *Sphingobacterium* encompasses at least 58 other species, including the close relatives of *S. multivorum*, namely *S. siyangense* and *S. spiritivorum* (type strain of the genus) [19,20,21,22]. Moreover, since its original description, several ‘environmental’ *S. multivorum* strains have been added to the species [4,6], and—following a search for strains related to our strain w15—we recently came across a highly interesting strain, BIGb0170 [23].

In recent years, bioinformatics-based genome metrics have developed into the standard for delineating bacterial species [24]. In particular, average nucleotide identity based on the BLASTn algorithm (ANIb) and genome BLAST distance phylogeny (GBDP)-based digital DNA: DNA hybridization (dDDH) methods have become the methods of choice to determine species boundaries and confirm identities [25]. In addition, tetranucleotide frequency (TETRA) assessments can yield supporting information on bacterial relatedness [26]. However, in spite of the fuzziness of the current species designations around *S. multivorum*, such advanced genome-based metrics have not yet been applied to the respective species.

Based on the emerging information gathered so far, we hypothesized that strain w15 might either represent a new subclass within the species *S. multivorum* (potentially adding a novel ecological type to this species) or belong to a group that is closely associated with *S. multivorum*. Hence, we examine the available evidence (based on analyses of both molecular and experimental data) with respect to the taxonomic and ecophysiological position of strain w15 and comparator strains, including the *S. multivorum* type strain. The results of these analyses clearly demonstrate that strain w15 belongs to a novel clade that is associated with *S. multivorum* and *S. siyangense*, for which we propose the name *S. paramultivorum*.

## 2. Materials and Methods

### 2.1. Strains and Genomes

Strain w15 was used as the ‘start’ organism. Moreover, data on diverse strains of *S. multivorum*, including the type strains, were selected across strain collections: NCTC 11343^T^; DSM 11691^T^ (FDAARGOS 1203), FDAARGOS 1141 (DSM 11689, a.k.a. IFO 14087; NBRC 14087; F-179); FDAARGOS 1142 (DSM 11703), FDAARGOS 1140 (DSM 11688, KS 0433), FDAARGOS 1143 (DSM 15469, NCTC 11034), BIGb0170. In addition, the following close relatives of *S. multivorum* were also used: *S. siyangense* PDNC006, *S. siyangense* SY1^T^, *Sphingobacterium* sp. G1-14.

### 2.2. Cloning of Strain w15 16S rRNA Genes and Sequencing

Cells of strain w15 were recovered from −80 °C stocks and placed on tryptic soy agar (TSA, Sigma–Aldrich, Darmstadt, Germany) plates. After 24h incubation at 28 °C, a single colony was picked up and streaked to a new TSA plate, which was incubated at 28 °C for another 24 h. Afterwards, a single colony was introduced into 20 mL Luria-Bertani (LB, Sigma–Aldrich, Darmstadt, Germany) broth in a 100 mL Erlenmeyer flask. After 16h incubation at 28 °C, 1.8 mL of culture was harvested for DNA extraction using the DNeasy UltraClean Microbial Kit (Qiagen, GmbH, Hilden, Germany). Next, two sets of primers (B8F/515R and B8F/1492R) were used to generate 16S rRNA gene based amplicons by standard PCR [27]. The amplicons were then cleaned using the QIAquick PCR Purification Kit (Qiagen, GmbH, Hilden, Germany), and used for cloning in the pGEM-T Easy Vector Systems Kit (Promega, GmbH, Walldorf, Germany). The cloned amplicons were then subjected to sequencing at BaseClear (BaseClear B.V., Leiden, The Netherlands) using Sanger technology.

### 2.3. Detection of 16S rRNA Gene Types—And Their Ratio—In Transcriptomes

Three different sequence variants were found in the region between the 16S rRNA positions 186–210. These sequences (and their reverse complementary sequences) were used as proxies for (at least) three different 16S rRNA genes (Supplementary Material, Table S1) in order to assess their prevalence in RNA samples taken from a three-partner (strain w15, *Citrobacter freundii* so4, *Coniochaeta* sp. 2T2.1) wheat straw degradation experiment (Wang et al., in preparation). The sequences were not found in either *C. freundii* so4 or in *Coniochaeta* sp. 2T2.1. Other differences were found in the 16S rRNA gene region 463–476 (AAATACGTGTATTT and TTCTACGTGTAGGG). Different sequences found in this region were also used as proxies in a dichotomic scheme. The numbers of reads of each sequence type were summed and used for the ratio calculations, as given below. Three variants (denoted as C_GA, T_GA and T_CA) were found at positions 186–210, and two variants (denoted as AAA_TTT and TTC_GGG) were found in region 463–476. The ratio of a variant was calculated as, for example, ratio of variant C_GA = read count of variant C_GA/(sum of read counts of C_GA, T_GA and T_CA), ratio of AAA_TTT = read count of AAA_TTT/(sum of read counts of AAA_TTT and TTC_GGG).

### 2.4. Phylogenetic Analyses

Multiple sequence alignments were performed using the ‘Muscle’ default setting in the Mega 7 software [28]. The best DNA/Protein models (ML) were determined within this platform using partial deletion of gaps/missing data with 95% site coverage cutoff. Then, phylogenetic trees were inferred using maximum likelihood analyses [29] under the K2+G+I mode of evolution [28]. Bootstrap analyses were performed using 1000 resamplings to estimate the confidence values of the tree topologies [30].

To clarify the taxonomic position of w15, sequences of five conserved marker genes (*rplC*, *groL*, *ftsA*, *dnaE*, *gyrB*) were retrieved from NCBI (whole genome assemblies), and phylogenetic analyses were performed using the concatenated sequences in Mega 7 [28]. Each gene was first aligned independently using the ‘Muscle’ default setting. The best DNA/Protein models (ML) were determined using partial deletion of gaps/missing data with 95% site coverage cutoff [28]. The genes were then concatenated and aligned, and maximum likelihood phylogeny was determined under the LG + α + γ model of evolution and with 1000 bootstrap replicates.

### 2.5. Comparative Genome Analyses

ANI values and TETRA correlation coefficients were obtained using SpeciesWS [31]. The ANI analyses relied on the algorithms BLAST+ (ANIb) and MUMmer-Maximum Unique Matches (ANIm). Additionally, Tetra Correlation Search (TCS) analyses (shown as Z-values) were also done to provide a hit list for insights into the relationships with genomes of the reference genome database [31]. Typically, the ANI values between genomes of the same species are above ~95%, and so we used this threshold for species designations.

### 2.6. Genome-Based Taxonomic Analyses

Whole genome-based taxonomic analyses and dDDH were performed using the type strain genome server (TYGS, https://tygs.dsmz.de, accessed on 20 August 2021) [32] with the following methods.

***Determination of closely-related type strains***—Determination of the relatedness of type strain genomes was done in two complementary ways. First, ‘user’ genomes were compared against all type strain genomes available in the TYGS database via the rapid MASH algorithm [33], and then ten type strains with the smallest MASH distances were chosen per genome. Secondly, an additional set of ten closely-related type strains was determined via the 16S rRNA gene sequences. These were extracted from the user genomes using RNAmmer [34] and each sequence was subsequently BLASTed [35] against the 16S rRNA gene sequence of each of the currently 14,723 type strains available in the TYGS database. Distances calculated by formula d5 [25] were finally used to determine the ten closest type strain genomes for each of the user genomes.

***Pairwise comparison of genome sequences***—For the phylogenomic inference, pairwise comparisons across selected genomes were conducted using GBDP and accurate intergenomic distances inferred under the algorithm ‘trimming’ with distance formula d5 [25]. One hundred distance replicates were calculated for each. dDDH values and confidence intervals were calculated using the recommended settings of the GGDC 2.1 [25].

***Phylogenetic inference***—The resulting intergenomic distances were used to infer a balanced minimum evolution tree with branch support via FASTME 2.1.6.1 including SPR postprocessing [36]. Branch support was inferred from one hundred pseudo bootstrap replicates each. The trees were rooted at the midpoint [37] and visualized with PhyD3 [38].

***Type species-based species and subspecies clustering***—Type species-based species clustering using a 70% dDDH radius around 28 selected *Sphingobacterium* type strains was done as previously described [32]. Subspecies clustering was done using a 79% dDDH threshold as previously recommended [39].

## 3. Results

Although initial analyses—based on the then-available almost-full 16S rRNA gene sequence—had placed strain w15 within the species *S. multivorum* [17], questions arose with respect to its precise affiliation. Major issues were the (potential) microheterogeneity across the ribosomal operons, and close association only with the so-called ‘divergent’ 16S rRNA gene of the type strain. We thus first set out to examine the overall genomic features of a selection of relevant strains associated with *S. multivorum*. We then made an in-depth analysis of the strain w15 16S rRNA genes, based on detailed analyses of the microheterogeneity across such operons.

### 3.1. General Properties of the Genomes of Strains Assigned or Related to S. multivorum and S. siyangense

To shed light on the nature of the association of strain w15 with the species *S. multivorum*, key genome features were first examined. As comparators, the genomes of two *S. siyangense* strains (SY1^T^; PDNC006) and one *Sphingobacterium* sp., GI-14, were also examined (Table 1). All *S. multivorum* strain genomes (NCTC 11343^T^; NCTC 11034; DSM 11691^T^; FDAARGOS 1140; FDAARGOS 1143) showed G+C% of 40.0–40.1%, genome sizes of 5.7–6.0 Mbp, and had totals of 4879–5310 identifiable genes. The *S. siyangense* strains (SY1^T^; PDNC006) showed G+C% of 39.8%, genome sizes of 6.3–6.8 Mbp, and had totals of 5482–5741 identifiable genes. Regarding the genome features, strain w15 had a G+C% of 39.8%, a genome size of 6.68 Mbp, with a total of 5541 identifiable genes. Interestingly, it was very akin to a recently described strain, BIGb0170, which revealed a G+C% of 39.9%, a genome size of 6.43 Mbp, and a total of 5348 genes.

Across the board, and assuming an average bacterial gene size of about 1000 bp, relatively high gene loads and, consequently, relatively low levels of non-coding or non-assigned DNA were found. These were estimated to be around 83–89% (strains w15 and BIGb0170 83%, *S. siyangense* SY1^T^ 87% and *S. multivorum* NCTC 11343^T^ 89%) (Table 1). This indicated a moderate to strong control of genome quality via deletion bias in these organisms.

Interestingly, from the first analyses, we initially found evidence for the existence of 5–7 5S rRNA operons across all genomes, and 6–7 (all *S. multivorum* strains) versus just 1 (w15, and SY1^T^) 16S rRNA operon(s). This puzzling finding constituted the basis for our further investigations.

### 3.2. Unraveling the 16S rRNA Gene Features of Strain w15 and Other Strains

Here, we examine the 16S rRNA operons found across all *S. multivorum* strains, including the type strains NCTC 11343^T^ and DSM 11691^T^, with implications for the identity of strain w15. First, the available 16S rRNA gene sequence data (not shown) indeed indicated strain DSM 11691^T^ to be identical to NCTC 11343^T^, suggesting these are interchangeable (also including strain NBRC 14947^T^, a copy of NCTC 11343^T^). Hence, for further work, we used information gathered from strain NCTC 11343^T^, as the ‘canonical’ *S. multivorum* type strain. Remarkably, detailed analyses of this strain’s genome revealed the presence of seven ribosomal RNA operons (Table 1). Of these, six were largely identical (internal differences < 0.5%), with one being rather divergent. For the purpose of our study, the first group of six operons was coined the canonical group, and the outlier was coined the divergent operon.

#### 3.2.1. Small Regions in the 16S rRNA Gene V2 Region Drive the Microheterogeneity

Major differences between the canonical and the divergent 16S rRNA gene of *S. multivorum* NCTC 11343^T^ were found to be located in a small region (part of variable region 2 – V2) between positions 186 and 210 (13 differences/gap in a total of 25 base positions), as listed in Table 2. The remainder of the about 1500-bp sequence was rather similar (not shown). Thus, the divergent 16S rRNA gene sequence (UAUU01000005.1_rrna_60) had 98.89% similarity with the six canonical ones, while the similarities among the latter were 99.54–99.93% (Table 2). In this study, a random sequence from the canonical group was chosen to represent the canonical 16S rRNA gene (UAUU01000011.1_rrna_80) of *S. multivorum* NCTC 11343^T^.

Remarkably, the genome-derived 16S rRNA gene sequence of strain w15 turned out to be most similar (98.89%) to the divergent sequence present in *S. multivorum* strain NCTC 11343^T^, and lower similarities were found with the canonical ones (98.48–98.76%, data not shown). Expectedly, the differences across the w15 and NCTC 11343^T^ 16S rRNA gene sequences were—to a high extent–also driven by divergence in the 186–210 region (13 differences/gap in a total of 25 base positions), with one-base differences at just 8–9 positions over the remaining length (~1500 bases).

#### 3.2.2. Exploring the 16S rRNA Operons in Strain w15 and Related Strains

For strain w15, we PCR-generated 40 clones, all of which were fully sequenced (see Figures S1–S3, Table S1). In the 40 clones, differences were found at three positions (in different combinations): position 186: C or T (24 C, 14 T); positions 207–209: G-A or A-C (35 G-A, 4 A-C); positions 463–465/474–476: 23 AAA-TTT, 15 TTC-GGG. The 40 sequences were sorted into two major groups based on the differences at positions 463–465/474–476: AAA-TTT and TTC-GGG. We then examined how the differences at the other positions were distributed within these two groups. Results are summarized in Table 3. A total of six sequence types was found, with differences among these being < 1% (Table 4). We hypothesized these represent (partial) sequences of at least six 16S rRNA gene operons. Of these, five almost-full sequences from each type of 16S rRNA gene operon were used in later analyses (w15, seq 1 to seq 5, Table 4).

**Figure 1:**
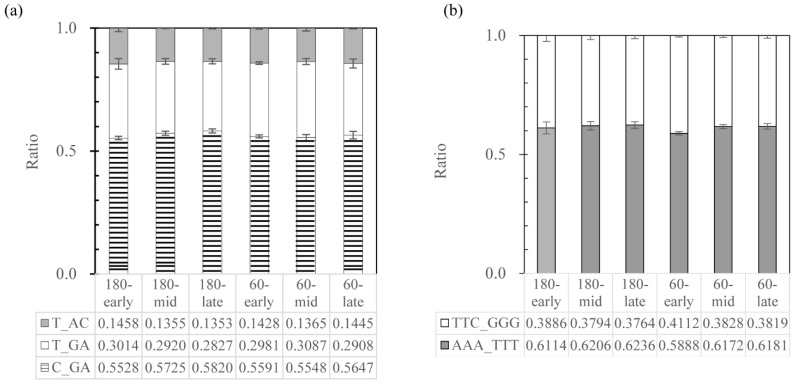
16S rRNA types of strain w15 (**a**) ratio of T_AC (grey)/T_GA (blank)/C_GA (zebra lines) at V2 region, and (**b**) ratio of TTC_GGG (blank)/AAA_TTT (grey) at V3 region. 180: samples from 180 rpm shake cultures; 60: samples from 60 rpm cultures; early: 24 h; mid: 5 days; late: 13 days.

To examine whether the different 16S rRNA gene copies play different roles in gene expression in the biodegradative consortia, the ratios of different 16S rRNA types of strain w15 growing in a three-partner consortium (Wang et al. in prep) were evaluated; these were calculated separately in the V2 and V3 regions (due to the technical limitation). Samples from three time points (early-24h, mid-5 days, and late-13 days) obtained from two shaking speeds (60 and 180 rpm) were tested. The ratio of variants T_AC/T_GA/C_GA (V2 region) and variants AAA-TTT/TTC-GGG (V3 region) were found to be stable over time and shaking speed, being around 1:2.1:4.0 and 1.6:1 respectively (Figure 1), similar to the ratios detected by the cloning and sequencing effort (Table 3).

Analysis of the 16S rRNA sequences within each strain with the different types of 16S rRNA sequences (labelled seq 1, seq 2…; Table 4) revealed all seven 16S rRNA gene sequences of strain BIGb0170 to diverge < 0.2% across each other. Moreover, none of these seven sequences had >99% similarity with either the divergent or canonical 16S rRNA gene sequences of *S. multivorum* NCTC 11343^T^ or *S. siyangense* SY1^T^. In contrast, all seven were >99% similar to sequences of strain w15, with the main differences being found at positions 186, 207–209, and 463–465/474–476. This resembled the variances found within strain w15 (Table 4), and indicated that strains BIGb0170 and w15 are very closely related.

We further found all seven 16S rRNA gene operons of strain FDAARGOS 1141 to have differences < 1%, with none of them having >99% similarity with either the divergent or the canonical 16S rRNA genes of *S. multivorum* NCTC 11343^T^. Instead, these had >99% similarity with the strain w15 16S rRNA gene sequences and one had 99.4% similarity with *S. siyangense* SY1^T^ (Table 4).

However, the situation was complex for *S. siyangense* (strain PDNC006), as it showed <1% difference among all seven 16S rRNA gene operons, with two operons being similar to the canonical 16S rRNA gene of strain NCTC 11343^T^ (99.48% similarity), and the other five having <1% difference with the similar ones of *S. siyangense* SY1^T^. An overview of these differences and similarities can be found in Table 4.

### 3.3. Phylogenetic Analyses

**Figure 2:**
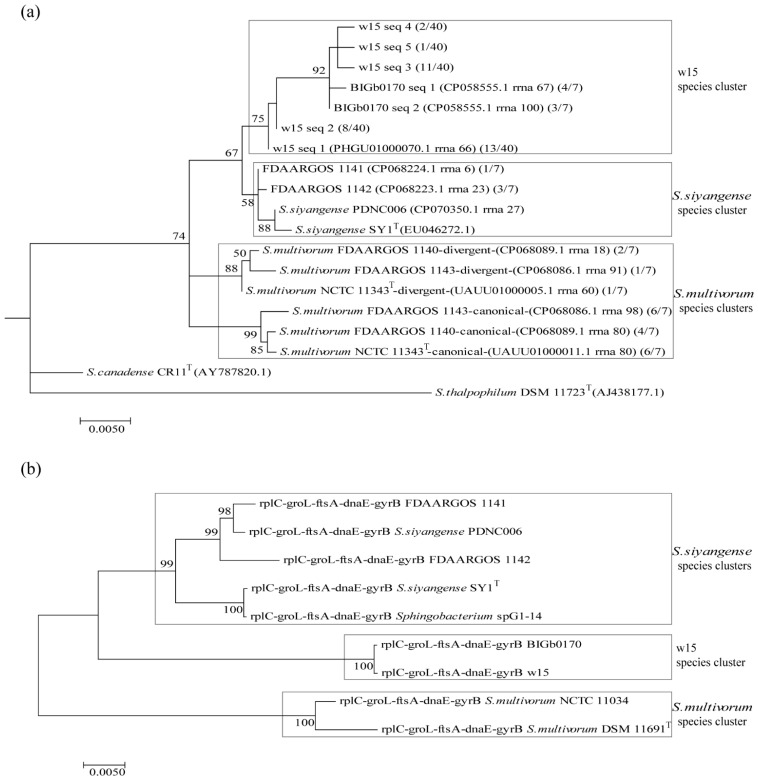
Phylogenetic tree of strain w15, BIGb0170, *S. multivorum* NCTC 11343^T^, *S. siyangense* SY1^T^ and related species, based on (**a**) 16S rRNA and (**b**) five concatenated core genes (*rplC*, *groL*, *ftsA*, *dnaE*, *gyrB*).

The analyses based on alignment of the 16S rRNA genes thus showed that the divergent and canonical 16S rRNA gene sequences of *S. multivorum* NCTC 11343^T^ clustered into two groups (Figure 2a). Clearly, strains w15, BIGb0170, FDAARGOS 1141 and FDAARGOS 1142 were separate from these *S. multivorum* clusters. Whereas the sequences of the latter strains showed potential intermixing with *S. siyangense* SY1^T^, those of strains w15 and BIGb0170 revealed grouping within a separate cluster.

To further examine these uncertain placements, we constructed a five-gene concatenate based gene tree (Figure 2b). Analysis of the similarities of the core genes *rplC*, *groL*, *ftsA*, *dnaE*, *gyrB* showed that the w15 genes had high (>98.7%) amino acid similarities with the relevant counterpart sequences of all tested strains (Table S2), with the highest (100%) similarities with the respective genes of strain BIGb0170 (Table S2). However, at the DNA level, the five-gene concatenate trees clearly showed the existence of three groups among the selected strains: group 1—*S. multivorum* (DSM 11691^T^/NCTC 11343^T^, NCTC 11034); group 2—strains w15 and BIGb0170; group 3—*S. siyangense* (SY1^T^, PDN006). Interestingly, strains FDAARGOS 1141 and FDAARGOS 1142 both clustered tightly into group 3 (*S. siyangense*).

### 3.4. Average Nucleotide Identity (ANI) and Tetranucleotide Frequency (TETRA) Analyses

**Figure 3:**
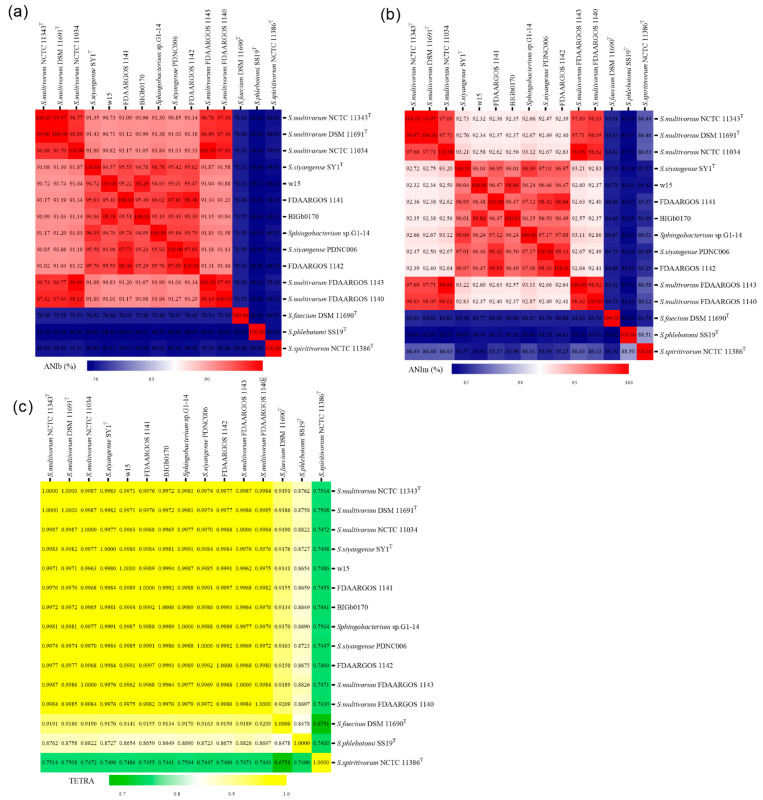
Heat maps of average nucleotide identities; (**a**) ANIb, (**b**) ANIm; and tetranucleotide frequencies (**c**) TETRA analyses of strains w15, BIGb0170, *S. multivorum* NCTC 11343^T^, *S. siyangense* SY1^T^ and related species. Selected ANIb (> 95%) and TETRA (> 0.999) values were used as species thresholds.

To further clarify the identities of strains w15, BIGb0170, FDAARGOS 1141 and FDAARGOS 1142, we calculated the ANI and TETRA values between the genomes of these four strains, next to 11 other ones, together belonging to six related *Sphingobacterium* species. The data indicated that the type strains of *S. multivorum* (NCTC 11343^T^ and DSM 11691^T^) were virtually identical, with ANIb, ANIm and TETRA values being 99.97%, 99.97% and 1.0000, respectively (Figure 3). Furthermore, the only other strains denoted as *S. multivorum* that passed both the ANIb and ANIm species thresholds (>95%), were NCTC 11034, FDAARGOS 1140 and FDAARGOS 1143 (a.k.a. NCTC 11034, DSM 15469) (Figure 3). However, these did not pass the TETRA species thresholds. With respect to strain w15, it appeared as part of a small group including strain BIGb0170 (and FDAARGOS 1141), with >95% ANIb relatedness between them (Figure 3a), versus < 95% with the other strains including *S. multivorum*. This grouping was confirmed by the ANIm values (Figure 3b), albeit at higher relatedness levels. The TETRA values (Figure 3c) were largely in line with this contention, with strain w15 having 0.9994 similarity with BIGb0170 (but lower with FDAARGOS 1141, examined further in the next section), versus TETRA levels of <~0.9981 with both the *S. multivorum* and *S siyangense* (type) strains.

#### 3.4.1. Taxonomic Placement of FDAARGOS 1141 and FDAARGOS 1142

Surprisingly, the genomes of strains w15, BIGb0170, FDAARGOS 1141 and FDAARGOS 1142 only showed 91–92% ANIb and ANIm values to *S. multivorum* NCTC 11343^T^. This provided strong evidence for the tenet that these four strains fall outside of the species *S. multivorum*. Furthermore, only strains FDAARGOS 1141 and FDAARGOS 1142 showed ANIb (and ANIm) values > 95% with *S. siyangense* SY1^T^, whereas strains w15 and BIGb0170 had ANIb values of only 94.57% and 94.78, respectively (Figure 3). In contrast, FDAARGOS 1141 and FDAARGOS 1142 both failed the TETRA species threshold with *S. siyangense* SY1^T^, as both revealed values of 0.9984; this was also found above (*S. multivorum* species). Based on these data, we consider the placement of FDAARGOS 1141 and FDAARGOS 1142 into a (sub)group related to *S. siyangense* to be commendable; however, this is to be examined further.

#### 3.4.2. Proposal of a New Species Based on Strains w15 and BIGb0170

Clearly, the genomes of w15 and BIGb0170 were highly similar, with ANIb and ANIm values (98.3% and 98.9% between them) exceeding the respective species thresholds (>95%). Remarkably, as discussed in the foregoing, the two genomes also passed the TETRA species cutoff value (>0.999), being 0.9994 (Figure 3). Finally, the core gene concatenate comparisons (Figure 2b) were consistent with the high relatedness of the genomes of these two strains. Thus, the collective evidence points to the conclusion that strains w15 and BIGb0170 form a taxonomically tight group, which differs substantially from *S. multivorum* and *S. siyangense*, and establishes a novel species, for which the name *Sphingobacterium paramultivorum* is proposed.

### 3.5. Genome-Based Taxonomic Analyses

**Figure 4:**
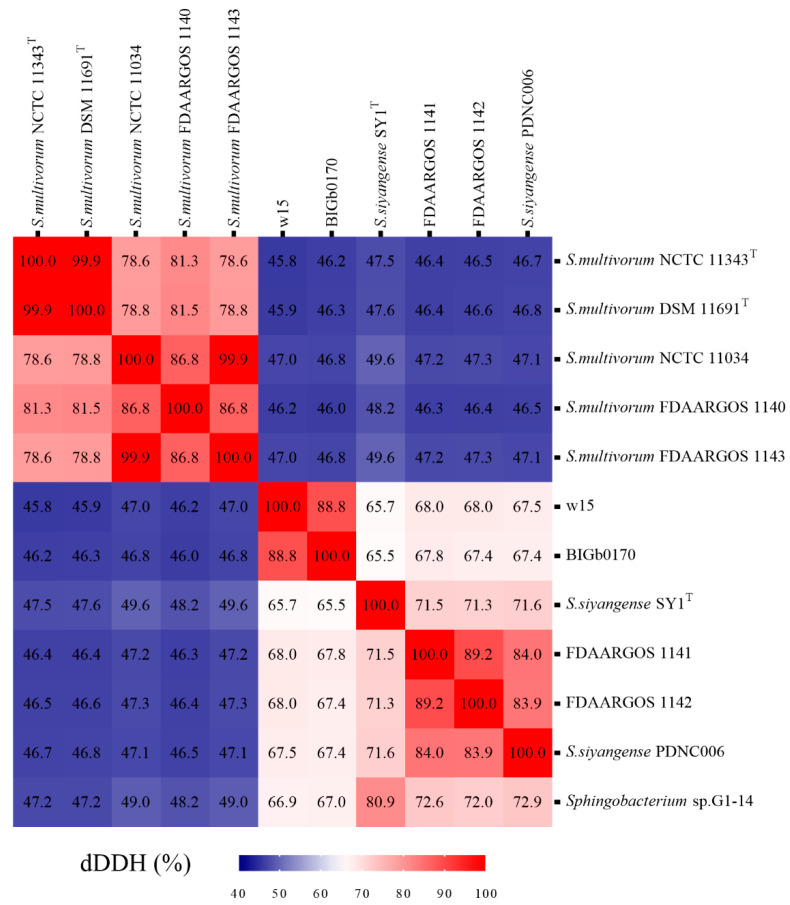
Heat map of dDDH analyses of strain w15, BIGb0170, *S. multivorum* NCTC 11343^T^, *S. siyangense* SY1^T^ and closely related species.

**Figure 5:**
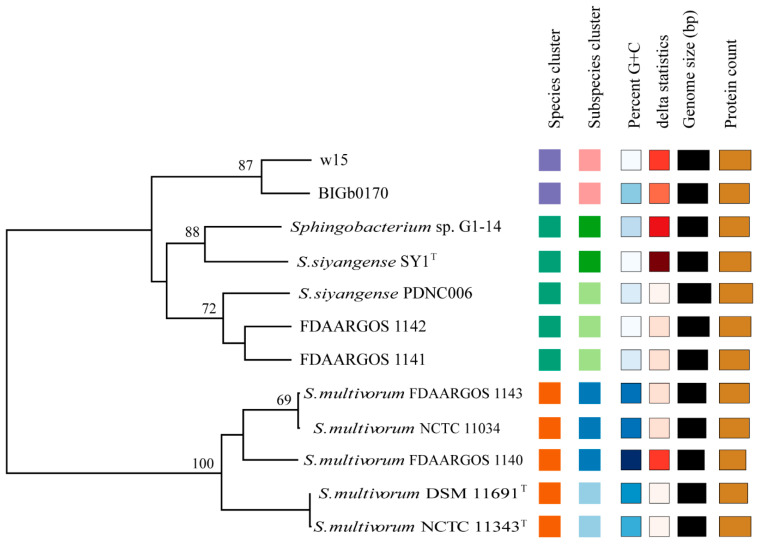
Tree inferred with FastME 2.1.6.1 [36] from GBDP distances calculated from the genome sequences. The branch lengths are scaled in terms of GBDP distance formula d5. The numbers above the branches are GBDP pseudo-bootstrap support values > 60% from 100 replications, with an average branch support of 65.2%. The tree was rooted at the midpoint [37]. Colored squares indicate relatedness (same color, same parameter value class, on the basis of standard FastME settings).

Using the cutoff value of dDDH (>70%), the aforementioned genomes were analyzed by digital hybridization analyses. The data showed that the genomes of strains w15 and BIGb0170 both had dDDH values of <70% with those of the *S. multivorum* and *S. siyangense* type strains (being 46% and 66%, respectively). Overall, the genomes clustered into three groups (Figure 4), i.e., the predicted *S. multivorum* and *S. siyangense*, and the newly proposed *S. paramultivorum* (w15 and BIGb0170) groups. Similarly, the genomes of both FDAARGOS 1141 and FDAARGOS 1142 had dDDH values of 46–47% with that of *S. multivorum* NCTC 11343^T^ (below the species boundary) and 71–72% with that of *S. siyangense* SY1^T^. The clustering was further consistent with the five-gene phylogenetic tree and the ANI analyses. A 79% dDDH threshold was used by us for further (subspecies) clustering, which resulted in two subspecies identified under *S. multivorum* and *S. siyangense* (Table 5, Figure 5); this was in line with the five-gene phylogenetic tree and the TETRA analysis.

Furthermore, the whole genome-based taxonomic analyses using the type strain database (TYGS) confirmed that strains FDAARGOS 1140, FDAARGOS 1143 and NCTC 11034 all belong to the species *S. multivorum*, and strain PDNC006 to *S. siyangense* (Table 5). The analysis also confirmed the conclusion that strains w15 and BIGb0170, together, form a new species, and FDAARGOS 1141 and FDAARGOS 1142 are highly related to *S. siyangense* (Table 5).

### 3.6. Examination of Strain Origins and Ecophysiological Data

In an attempt to determine the ecological ‘niches’ of the selected strains, all strain descriptions were examined with respect to the strain (ecophysiological) metadata. For strain w15, we used data generated in our own laboratory [17]. Table 6 shows a summary of these analyses. Clearly, two major types of strain origin were found: clinical or environmental (soil, nematode-associated or rotting plant matter) settings. Moreover, one may discern the glimpses of two types of ecological behavior, as it is likely the strains first found in clinical settings are often involved in infectious processes (interactions with hosts leading to infection), whereas those found in environmental settings often revealed involvement in biodegradative processes. Strain w15 is strongly involved in WS degradation, whereas the sister strain BIGb0170 is a context-dependent colonizer of *Caenorhabditis elegans*, in which host biodegradative processes also play roles. Clearly, the evidence further shows that strains of all three clusters are able to utilize a plethora of carbon sources, such as D-xylose, maltose, D-melibiose, D-fructose, D-glucose, sucrose, D-galactose, trehalose, lactose, cellobiose, melezitose, salicin, and N-acetyl-D-glucosamine.

Other traits differentiate the three clusters, as follows. Only *S. siyangense* SY1^T^ was able to grow at 4 and 42 °C, and survive at low pH (<4.0); it performs hydrolysis of L-arginine, D-sorbitol, L-sorbose, D-mannitol, xylitol, adonitol and glycerol. In contrast, neither w15 nor *S. multivorum* NCTC 11343^T^ were able to grow at 4 °C, 42 °C, pH 4.0 or utilize these carbon sources. Remarkably, all strains from the clinic (*S. multivorum* NCTC 11343^T^, NCTC 11034) were able to hydrolyse Tween 80, while w15 and BIGb0170 and *S. siyangense* SY1^T^ were not. *S. multivorum* NCTC 11343^T^ was not able to utilize D-mannose or L-rhamnose, but w15 and *S. siyangense* SY1^T^ were.

## 4. Discussion

In this study, we examined the available data on the genomic, phylogenetic and ecophysiological traits of selected relevant *S. multivorum* and related *Sphingobacterium* strains, including the biodegradative strain w15 (Table 1). Following this data gathering exercise, we placed a focus on key data that assist us in the elucidation of the taxonomic placement of the relevant strains w15, BIGb0170 and several FDAARGOS ones, which so far have been associated loosely with *S. multivorum*. Indeed, following the coining of the genus *Sphingobacterium* by Yabuuchi et al. in 1983 [18], there have been many additions to this genus, including the species *S. multivorum*. However, the exact placement of *S. multivorum* has been under debate and the position of several related organisms has also remained unclear.

***Unraveling the taxonomic identity of selected strains associated with* S. multivorum—*16S rRNA gene features***—Clearly, the fact that multiple 16S rRNA operons occur within and across the selected *Sphingobacterium-* strains, including the *S. multivorum* type strain, poses key problems for the taxonomic consistency in these organisms. This facet also has strong implications for the taxonomic identity of strain w15, which was previously assigned to *S. multivorum* based on just a single 16S rRNA gene sequence [17]. In addition, previous studies revealed the species *S. multivorum* to be very close to *S. siyangense* [20], and these two species were often noticed to cluster together in 16S rRNA sequence based phylogenetic trees [19,20,44]. Thus we surmised that a “species cluster” (<1% divergence at the 16S rRNA gene sequence level) might be present. However, this tenet appeared to be insufficient to delineate species, as in species containing multiple 16S rRNA gene copies, sequences with up to 1% divergence or more can occur (Table 4). The current way of categorizing bacteria into species by 16S rRNA gene sequence identity would unavoidably classify a great diversity of phylogenetically different bacteria into the same species [45]. To the best of our knowledge, this is the first report on multiple 16S rRNA sequences with such high (1% or more) divergence within one single strain. The data obtained in this study indicate that a complex 16S rRNA gene sequence picture is present across all strains. In fact, we present evidence for the contention that strain w15 has at least six 16S rRNA genes, with microheterogeneity between them. Moreover, this situation was akin to that in strain BIGb0170 and also to that of the species *S. multivorum,* although it was different by sequence. In detail, a highly variable 16S rRNA gene region—position 186–210—was identified as being mainly responsible for the differences, whereas another region—position 463–465/474–476—was also important. The use of the sequence divergences in these two regions in rRNA expressed under different conditions and over time in degrader consortia, however, did not reveal differences in the ratio of the different rRNA types (Figure 1). This debunked our hypothesis of an ecophysiological driver for the existence of the multiple diverse 16S rRNA gene copies, at least within the limits of the experimental contexts.

***The differentiation of the selected strains into three groups is robust and supported by several lines of evidence***—Although the 16S rRNA gene analyses were blurred by multiple sequences and sequence microheterogeneities, at least three lines of evidence provided solid evidence for the separation of strains w15 and BIGb0170 from *S. multivorum* and *S. siyangense*, and their placement together into a novel species. These were, first, the solid evidence yielded by ANIb (and ANIm), in which the w15/BIGb0170 group stood out as clearly separate from the other groups, passing the species threshold set at 95% (Figure 3a,b). This was supported by the following TETRA analyses, in which a threshold of 0.999 was used (Figure 3c). The second thread was provided by the dDDH and whole genome based taxonomic analyses; the <70% dDDH values with the *S. multivorum* and *S. siyangense* comparator genomes point to a new species formed by w15 and BIGb0170 (Figure 4 and Figure 5, Table 5). The third thread came from the five gene-concatenate analyses, which clearly showed the existence of three clusters, with strains w15 and BIGb0170 clustering closely together. Overall, we consider these analyses to provide very convincing evidence for the contention that strains w15 and BIGb0170 together constitute a novel species, coined *Sphingobacterium paramultivorum*, which is related to *S. siyangense* and *S. multivorum*.

***Habitats, niche and ecological versatility of the selected Sphingobacterium strains***: among all selected strains, several, including w15, BIGb0170, FDAARGOS 1141 (from soil) and FDAARGOS 1142 (from activated sludge) came from environmental samples, whereas others, including *S. multivorum* NCTC 11343^T^ and NCTC 11034 were from clinical specimens. This shows the divergent environmental habitats in which the organisms that were loosely associated with *S. multivorum* can be found. It is logical to posit that the organisms in this broad group can thrive in environments in which pulses of nutrients become available at times, establishing conditions for rapid growth provided a quick start (kick-off) can be made. Previous studies have shown that *S. multivorum* strains can thrive in various environments, from plant polymers [4,5] to hexaconazole triazole/petroleum hydrocarbon contaminated soil [8,9,10,11]. One can postulate that such ecological situations can and will arise in both the environmental and clinical settings, establishing suitable niches and driving the growth and evolution of the niche occupants. Given that the strains studied were obtained from soils, plant waste, plastics, and clinical settings (Table 6), we posit that their primary ‘habitat’ might be broad. What does this mean for the ecological niche they occupy, and can we discern differences in this niche occupancy across the three groups we identified? Defining niche as the combination of conditions, energy generation and nutritional parameters, yielding a multiple-axis ecological space area, it appears difficult to affirm the precise niche (and habitat) space occupied by the *S. multivorum* associated organisms. Clearly, based on their (shared) genome traits, it appears that all organisms can thrive in carbon- and oxygen-rich settings, where growth depends on oxidative processes. In the respective habitats, it is thought that pH and temperature are drivers that possibly define the (sub)niches, with obvious differences between environmental and clinical settings (Table 6).

Given the above arguments, the situation with respect to the ecophysiological characteristics of the w15/BIGb0170 group, and its differentiation from the other two groups, is not clear and subject to speculation. Whereas strain w15 was selected by WS into a WS degradation process and so may find its niche there; BIGb0170 is adapted to life within nematodes (where conditions of biopolymer degradation may reign as well). A commonality is the soil origin of both organisms, in which conditions of fluctuating temperature, pH and nutrient levels commonly reign. Furthermore, from the genomic analyses plus some of the physiological tests, it appears that strains w15 and BIGb0170 are both quick responders to the appearance of carbonaceous substrate [3,23]. Together, such commonalities are consistent with the genome-based arguments for the placement of w15 and BIGb0170 into a new species, in which they might even represent different ecotypes. In contrast, we lack evidence for a thorough consideration of the niches and ecophysiological traits of the members of the other two groups.

***Can the nature of the genomes and the 16S rRNA genes across the selected sphingobacteria be linked to niche occupancy?*** All strains analyzed had relatively large genomes, with those of the w15/BIGb0170 group, together with those of the *S. siyangense* strains, having larger sizes than those of the *S. multivorum* group (Table 1). This could be explained by the contention that clinical *S. multivorum* group strains to some extent adapt to a host setting, having lost some functions that are important for survival in harsh (soil) settings [46]. Moreover, all genomes had relatively high gene loads (83–89%)—as is usual for bacterial genomes—which is usually taken as evidence for high gene conservation pressures due to regular events of selection, in the light of the commonly high deletion bias [46,47]. The genomes had two additional salient features, i.e., the ability to furnish the (secreted) enzymes for utilization of a plethora of different substrates, often derived from organic polymers, as carbon and energy sources, and the high number (up to seven) of 16S rRNA operons. The presence of multiple such operons, and the potential importance in the growth kick-off, was already reported for *Escherichia coli* [48,49] and some bacilli [50] but it was new for strain w15. In detail, strain w15 had at least six, slightly divergent, 16S rRNA gene operons, mainly driven by the highly variable 186–210 region. It indeed was a highly efficient grower in aerobic sequential batches with WS as the substrate, in which short growth periods, interspersed with periods of slow growth or growth arrest, were stimulated at the expense of WS as the carbon and energy source [3,17]. Indeed, in WS-driven consortia, strain w15 is—upon each introduction—confronted with a sudden flush of abundant resources, like the sugars (or organic acids) coming off the WS polymers hemicellulose and cellulose. Therefore, w15 will have to rapidly scale the transcriptional and translational apparatus up and down, and potentially this is reflected in ribosome numbers and also types of ribosomes (loaded with either the canonical or the divergent 16S rRNA). The fact that no changes in the ratios between different types were found (Figure 1) indicated the absence of strong selective pressures favoring one or another rRNA type, across the conditions (Wang et al., in prep). Thus, one may surmise that such operons (within the constraints of the study) are selected to a rather similar extent.

***The importance of multiple 16S rRNA operons for the ecology of strain w15***—As brought forward in the literature and the foregoing text, the resource availability in an environmental setting, being either high, moderate or low, is a key driver of the population dynamics of bacteria like strain w15. Thus, fluctuating resource concentrations result in particular temporal resource dynamics which likely selected—over evolutionary time—strains like w15 for optimal fitness [51]. Previous work has already shown that the number of rRNA gene copies may predict an organism’s growth rate as well as growth efficiency as affected by resource availability [51,52]. Thus, in light of the tenet that multiple rRNA operons go with possibilities for high (initial) growth rates, the rRNA gene copy numbers may dictate not only the growth rate, but also the speed of response to growth condition changes, in particular upshifts [48,52,53]. The rapid bacterial growth observed by us for strain w15 (Wang et al. in prep), may require a substantial instantaneous increase in cellular ribosome numbers (as compared to slow growth or growth arrest). Clearly, such multiple rRNA gene copies may support the rapid build-up of sufficient ribosome numbers, simply by facilitating rRNA transcriptions. To what extent the microheterogeneities across the different gene copies in w15 have effects is so far unclear, as the data reported here (Figure 1) appear to show relative constancy of the type ratios. Similar reasoning may apply to the other multi rRNA copy organisms studied here.

## 5. Conclusions

Altogether, it can be concluded from this study that there is consistency in the data which unequivocally show that strains w15 and BIGb0170 represent a new species, for which the name *Sphingobacterium paramultivorum* is proposed. The type strain will be *S. paramultivorum* w15, deposited at DSMZ under number DSM 106342. Briefly, it is characterized as: Gram-negative, non-motile, non-spore forming, rod-shaped, forming yellowish colonies; growth in aerobic conditions, in a narrow pH range across neutral pH (5.0–9.0, optimal at pH 7.0), mesophilic temperature range (20–30 °C, optimal at 28 °C), and moderate NaCl levels (0–1%); able to grow on multiple sugar compounds (α-methyl-D-glucoside, α-D-lactose, D-cellobiose, α-methyl-D-glucoside, N-acetyl-D-glucosamine, pectin, dextrin, inulin), but not on acid compounds (L-phenylalanine, i-erythritol, D-galacturonic acid, D-galactonic acid lactone, putrescine, L-serine, D-mannitol, L-asparagine, L-threonine, glycogen, itaconic acid).

## Figures and Tables

**Table 1:** Genome information of strains used in this study.

Strain	Genome Size (Mb)	GC%	Total Genes	Protein Encoding Genes	Pseudo Genes	5S rRNA	16S rRNA	23S rRNA	tRNA	RefSeq Assembly Accession	Gene Load (%)
w15	6.68	39.8	5541	5426	40	7	6 ^a^	1 *	63	GCF_009660355.1	82.95
BIGb0170	6.43	39.9	5348	5222	19	7	7	7	83	GCF_014109745.1	83.17
*S.multivorum * NCTC11343^T^	5.98	40.0	5310	4685	517	7	7	8	83	GCF_900457465.1	88.80
*S.multivorum * NCTC11034	6.01	40.1	5207	5050	53	6	6	6	83	GCF_900457115.1	86.64
*S.multivorum* DSM11691^T^	5.97	40.0	5126	4944	75	7	7	7	83	GCF_016894225.1	85.86
*S.multivorum* FDAARGOS 1140	5.66	40.1	4879	4718	50	7	7	7	87	GCF_016726045.1	86.20
*S.multivorum * FDAARGOS 1143	6.02	40.1	5221	5062	50	7	7	7	85	GCF_016725865.1	86.73
FDAARGOS 1141	6.29	39.8	5274	5156	11	7	7	7	83	GCF_016725925.1	83.85
FDAARGOS 1142	6.55	39.8	5527	5402	18	7	7	7	83	GCF_016725465.1	84.38
*S. siyangense * SY1^T^	6.29	39.8	5482	5330	79	5	– ^b^	–	65	GCF_007830445.1	87.15
*S. siyangense* PDNC006	6.83	39.8	5741	5607	27	7	7	7	83	GCF_016919365.1	84.06
*Sphingobacterium* sp. G1-14	6.33	39.8	5388	5235	46	7	7	7	83	GCF_002196555.1	85.12

^a^: Five kinds of 16S sequences, revealed from cloning (this paper) were added to the analyses; *: the one-copy 23S rRNA gene is based on preliminary data. ^b^: 16S rRNA gene diversity was not found from the genome assembly; EU046272.1 partial 16S rRNA sequence initially used for analysis was supplemented with information reported here. –: not found in the genome assembly.

**Table 2:** 16S rRNA gene sequence similarity within *Sphingobacterium multivorum* NCTC 11343^T^.

				Similarity to	
Strain	16S rRNA Designation	Accession no.	Sequences at Position 186–210	Canonical	Divergent
	divergent (1/7)	UAUU01000005.1_rrna_60	CAT--A-A-TTCTCCGGCATCGGAGTATT	98.89	100.00
*S. multivorum*		UAUU01000002.1_rrna_32	CAT**CA**A**C**A**G**TTC**G**C**AT**G-**T**TC-G-GT-T**G**	99.61	99.02
		UAUU01000009.1_rrna_17	CAT**CA**A**C**A**G**TTC**G**C**AT**G-**T**TC-G-GT-T**G**	99.93	98.96
NCTC 11343^T^		UAUU01000009.1_rrna_23	CAT**CA**A**C**A**G**TTC**G**C**AT**G-**T**TC-G-GT-T**G**	99.54	98.96
	canonical (6/7)	UAUU01000011.1_rrna_80	**T**AT**CA**A**C**A**G**TTC**G**C**AT**G-**T**TC-G-GT-T**G**	100.00	98.89
		UAUU01000002.1_rrna_47	CAT**CA**A**C**A**G**TTC**G**C**AT**G-**T**TC-G-GT-T**G**	99.74	98.76
		UAUU01000009.1_rrna_1	CAT**CA**A**C**A**G**TTC**G**C**AT**G-**T**TC-G-GT-T**G**	99.77	98.63

**Table 3:** Different 16S rRNA gene sequences in strain w15.

Ratio	Position 186	Position 207–209	Position 463–465—474–476	Cloning No. (1367 bp)
11/40	C	G-A	AAA-TTT	5, 9, 12, 15, 29, 30, 32, 33, 34, 38, 39
8/40	T	G-A	AAA-TTT	3, 4, 7, 10, 11, 21, 28, 35
3/40	T	A-C	AAA-TTT	8, 14, 16
11/40	C	G-A	TTC-GGG	1, 2, 18, 22, 24, 25, 26, 27, 31, 36, 37
2/40	T	G-A	TTC-GGG	20, 23
1/40	T	A-C	TTC-GGG	40

**Table 4:** 16S rRNA sequence similarities across *Sphingobacterium* strains, referring to w15 rrna_66.

			V2 Region	V3 Region	Similarity to			
Strain	16S Designation	Accession number	186–210 bp	463–465/ 474–476 bp	w15	NCTC Canonical	11343^T^ Divergent	SY1^T^
	seq 1 (11/40)	PHGU01000070.1_rrna_66	**C** ATATCTGACCGGCATCGGTT **G** G **A** T	AAA/TTT	100.00	98.69	98.89	99.06
	seq 2 (8/40)	cloning 28	**T** ATATCTGACCGGCATCGGTTGGAT	AAA/TTT	99,93	98.69	98.76	99.12
w15	seq 3 (11/40)	cloning 36	CATATCTGACCGGCATCGGTTGGAT	TTC/GGG	99,49	98.10	98.32	98.83
	seq 4 (2/40)	cloning 23	**T** ATATCTGACCGGCATCGGTTGGAT	TTC/GGG	99.34	98.10	98.17	98.68
	seq 5 (1/40)	cloning 40	**T** ATATCTGACCGGCATCGGTT **A** G **C** T	TTC/GGG	99.27	98.10	98.25	98.90
	seq 2 (3/7)	CP058555.1_rrna_100	**T** ATATCTGACCGGCATCGGTTGGAT	TTC/GGG	99.54	98.37	98.43	98.72
BIGb0170	seq 1 (4/7)	CP058555.1_rrna_67	CATATCTGACCGGCATCGGTT **A** G **C** T	TTC/GGG	99.48	98.24	98.50	98.92
*S. multivorum*	canonical (6/7)	UAUU01000011.1_rrna_80	**T**AT**C**A**A**C**A**G**TT**C-GCAT-G**T**T**C**GG**T**T**G**	AAA/TTT	98.69	100.00	98.89	98.32
NCTC 11343^T^	divergent (1/7)	UAUU01000005.1_rrna_60	CATA**AT**T**CT**CCGGCATCGG-**A**G**T**A**T**T	AAA/TTT	98.89	98.89	100.00	98.86
*S. siyangense* SY1T		EU046272.1	CATATCTGACCGGCATCGGTT **A** G **C** T	AAG/TTC	99.06	98.32	98.86	100.00
*S. siyangense*	(5/7)	CP070350.1_rrna_27	CATATCTGACCGGCATCGGTT **A** G **C** T	AGA/TCT	99.41	98.57	99.08	99.66
PDNC006	canonical (2/7)	CP070350.1_rrna_92	**T**AT**C**A**A**C**A**G**TT**C-GCAT-GTT**CA**G**T**T**G**	AGA/TCT	98.83	99.48	98.63	98.72
	seq 3 (1/7)	CP068224.1_rrna_6	CATATCTGACCGGCATCGGTTGGAT	AGA/TCT	99.67	98.76	98.95	99.40
FDAARGOS 1141	seq 2 (2/7)	CP068224.1_rrna_91	CATAT **G** TGACCGGCATCGGTTGGAT	TTC/GGG	99.28	98.50	98.76	98.99
	seq 1 (4/7)	CP068224.1_rrna_64	CATAT **G** TGACCGGCATCGGTTGGAT	TTC/GGG	99.28	98.50	98.76	98.99
	seq 2 (3/7)	CP068223.1_rrna_23	CATAT **G** TGACCGGCATCGGTTGGAT	AGA/TCT	99.54	98.76	99.02	99.40
FDAARGOS 1142	seq 1 (4/7)	CP068223.1_rrna_64	CATATCTGACCGGCATCGGTT **T** GAT	TTC/GGG	99.28	98.37	98.63	98.92
	W-like (1/7)	CP068089.1_rrna_11	**T** ATATCTGACCGGCATCGGTT **A** G **C** T	AAA/TTT	99.22	99.02	99.15	99.06
*S. multivorum*	divergent (2/7)	CP068089.1_rrna_18	CATA**AT**T**CT**C**T**GGCATCGG-**A**G**T**A**T**T	AAA/TTT	98.76	98.83	99.87	98.72
FDAARGOS 1140	canonical (4/7)	CP068089.1_rrna_80	**TATC**A**A**C**A**G**TT**C-GCAT-G**T**T**C**GG**T**T**G**	AAA/TTT	98.63	99.80	99.09	98.52
*S. multivorum*	divergent (1/7)	CP068086.1_rrna_91	CATA**AT**T**CT**C**T**GGCATCGG-**A**G**T**A**T**T	AAA/TTT	98.82	99.02	99.67	98.52
FDAARGOS 1143	canonical (6/7)	CP068086.1_rrna_98	CAT**C**A**A**C**A**G**TT**C-GCAT-G**T**T**C**GG**T**T**G**	AAA/TTT	98.69	99.67	98.83	98.26

**Table 5:** Taxonomic identification based on whole genome sequences.

Strain	Conclusion	Identification Result	Species Cluster	Subspecies Cluster
w15	proposed new species	*Sphingobacterium paramultivorum*	3	2
BIGb0170	proposed new species	*Sphingobacterium paramultivorum*	3	2
*S. multivorum* NCTC 11343^T^	belongs to known species	*Sphingobacterium multivorum*	2	1
*S. multivorum* DSM 11691^T^	belongs to known species	*Sphingobacterium multivorum*	2	1
*S. multivorum* NCTC 11034	belongs to known species	*Sphingobacterium multivorum*	2	0
*S. multivorum* FDAARGOS 1140	belongs to known species	*Sphingobacterium multivorum*	2	0
*S. multivorum* FDAARGOS 1143	belongs to known species	*Sphingobacterium multivorum*	2	0
*S. siyangense* SY1^T^	belongs to known species	*Sphingobacterium siyangense*	1	4
*Sphingobacterium* sp. G1-14	belongs to known species	*Sphingobacterium siyangense*	1	4
FDAARGOS 1141	belongs to known species	*Sphingobacterium siyangense*	1	3
FDAARGOS 1142	belongs to known species	*Sphingobacterium siyangense*	1	3
*S. siyangense* PDNC006	belongs to known species	*Sphingobacterium siyangense*	1	3

**Table 6:** Strain origins and ecophysiological data.

Strains	*Sp*. w15	*Sp*. BIGb 0170	*Ss.* SY1^T^	*Sm.* NCTC 11343^T^	*Ss.* FDAARGOS 1141	*Ss.* FDAARGOS 1142	*Sm.* FDAARGOS 1140	*Sm. * NCTC 11034	*S*sp. G1-14	*Ss. * PDNC 006
**Isolation** **Source**	Decaying Wood	Rotting Apple ^h^	Farm Soil	Spleen	Soil	Activated Sludge	Succinoglycan	Blood	Soil	Plastic Debris
**Growth at:**										
**4 °C**	−		+	−	−	−	−	−		
**42 °C**	−		+	−	−	−	−	−		
**pH 4.0**	−		+	−				−		
**NaCl 5%**	−		−	−	+	−	−	−		
**Tween 80**	−	−	−	+		+	+	+		
**L-arginine**	−	−	+	−						
**D-sorbitol**	−		+	−						
**L-sorbose**	−		+	−						
**D-mannitol**	−	−	+	−						
**Xylitol**	−		+	−						
**Adonitol**	−		+	−						
**Glycerol**	−		+	−						
**D-mannose**	+		+	−				−		
**L-rhamnose**	+		+	−						
**α-Methyl-D-glucoside**	+	+		+						
**L-asparagine**	−	−								
**L-phenylalanine**	−	−								
**i-erythritol**	−	−	+							
**D-galacturonic acid**	−	−								
**D-galactonic acid lactone**	−	−								
**Putrescine**	−	−								
**L-threonine**	−	−		v						
**Glycogen**	−	−		v						
**Itaconic acid**	−	−								
**Pectin**	+					+	−			
**Dextrin**	+	−	+							
**Inulin**	+		+	v		+	+			
Key enzymes produced:										
			^a^	^b^	^c^	^c^	^c^	^d^		
References	[17]	[23]	[20]	[13,18,40,41]	[6,40]	[41,42]	[41,42]	[13,18]	[43]	
Traits shared by all three clusters:										
Gram-negative, aerobic growth, no motility, rod shaped cells										
Carbon sources used by all three clusters: D-xylose, maltose, D-melibiose, D-fructose, D-glucose, sucrose, D-galactose, trehalose, lactose, cellobiose, melezitose, salicin, N-acetyl-D-glucosamine										

+: positive results. −: negative results. v: results vary between references. Blank cells: no data available. ^a^ oxidase, catalase, β-galactosidase. ^b^ oxidase, catalase, β-galactosidase, N-acetyl-β-glucosaminidase, α-fucosidase, β-glucosidase, α-glucosidase, valine, arylamidase, cystine arylamidase, trypsin, chymotrypsin, α-mannosidase, lactosidase. ^c^ oxidase, catalase, β-galactosidase, N-acetyl-β-glucosaminidase, α-fucosidase, β-glucosidase, α-glucosidase, valine, arylamidase, cystine arylamidase, trypsin, chymotrypsin, α-mannosidase. ^d^ β-galactosidase, N-acetyl-β-glucosaminidase, α-L-fucosidase, β-glucosidase. ^h^ host *Caenorhabditis elegan*s. *Ssp*: *Sphingobacterium species. Ss*: *S. siyangense. Sm*: *S.multivorum. Sp*: *S. paramultivorum*.

## Data Availability

The datasets generated during and analyzed during the current study are available from the corresponding author upon reasonable request.

## Supplementary Materials

The following are available online at https://www.mdpi.com/article/10.3390/microorganisms9102057/s1; Figure S1: Difference could be due to B8F primer (AGAGTTTGATCMTGGCTCAG), Figure S2: Random differences, Figure S3: Differences with a clear ratio/frequency, Table S1: sequences used as probes for 16S rRNA ratio of w15, and Table S2: similarity of 9 strains to w15 of 5 protein genes (amino acid).
