# Supplementary material for: Considerations on the Identity and Diversity of Organisms Affiliated with Sphingobacterium multivorum—Proposal for a New Species, Sphingobacterium paramultivorum
Source: Microorganisms. 2021 Sep 29;9(10):2057. doi: 10.3390/microorganisms9102057 (PMC8540502; doi:10.3390/microorganisms9102057)
Supplement: Supplementary file 1 [file microorganisms-09-02057-s001.zip › microorganisms-1373676-supplementary.pdf]

microorganisms

Article

Considerations on the Identity and Diversity of Organisms Affiliated with *Sphingobacterium multivorum*—Proposal for a New Species, *Sphingobacterium paramultivorum*

Yanfang Wang [1,*], Jolanda K. Brons [1] and Jan Dirk van Elsas [1,*]

[1] Cluster of Microbial Ecology, Groningen Institute for Evolutionary Life Sciences, University of Groningen, Groningen, the Netherlands; j.k.brons@rug.nl (J.K.B)

*Correspondence: wang-yanfang@qq.com (Y.W); j.d.van.elsas@rug.nl (J.D.vE)

microorganisms

Supplementary Material

16S sequence diversity from cloning of strain w15:

How different are the sequences within 40 cloning sequences of w15?

The principle a difference is included or not is based on:

1. Is the difference due to the B8F primer? If yes, then exclude, if no, then we further look at the differences (see Figure S1). The "A" difference was excluded due to the "M" in primer B8F.

Query range 1: 1 to 60

```
Query        8    AGAGTTTGATCCTGGCTCAGGATGAACGCTAGCGGCAGGCCTAATACATGCAAGTCGGAC  67
Query_60058  1    ............................................................  60
Query_60064  1    ...........A................................................  60
Query_60052  1    ............................................................  60
Query_60055  1    ...........A................................................  60
Query_60061  1    ...........A................................................  60
Query_60059  1    ...........A................................................  60
Query_60056  1    ...........A................................................  60
Query_60054  1    ..T........A................................................  60
Query_60053  1    ...........A................................................  60
Query_60063  1    ............................................................  60
Query_60060  1    ...........A.............................................A..  60
Query_60057  1    ............................................................  60
Query_60066  1    ...........A.................................M..............  60
Query_60062  1    ...........A................................................  60
Query_60065  10            ...................................................  60
```

Figure S1 Difference could be due to B8F primer (AGAGTTTGATCMTGGCTCAG)

2 Is the difference random, or a clear frequency can be found? Random differences could be the results of PCR error or sequencing error, so random differences as shown in Figure. S2 were excluded for sorting 16s sequence for w15.

Query range 2: 61 to 120

```
Query        68   GGGATCCGTCGG-AGAGCTTGCTCGAAGACGGTGAGA-GTGGCGCACGGGTGCGTAACGC  125
Query_60087  1    ............-........................-......................  58
Query_60077  1    ............-........................-......................  58
Query_60081  1    ............-........................-......................  58
Query_60079  1     ...........-........................TR.....................  58
Query_60078  1    ............-.........C..............-......................  58
Query_60088  1     ...K.......A.R......................-......................  58
Query_60070  1    ............-........................-......................  58
Query_60083  1    ............-........................-......................  58
Query_60084  1    ....K.......-...R.K.....S............-......................  58
Query_60082  1    ............-............T.....KW....-......................  58
Query_60058  61   ............-........................-......................  118
Query_60064  61   ............-........................-......................  118
Query_60052  61   ............-........................-......................  118
Query_60055  61   ............-........................-......................  118
Query_60061  61   ............-............G...........-......................  118
Query_60059  61   ............-........................-......................  118
```

Figure S2 Random differences

Only differences with a clear ratio/ frequency were included for analysis. See the differences in red rectangle of Figure S3.

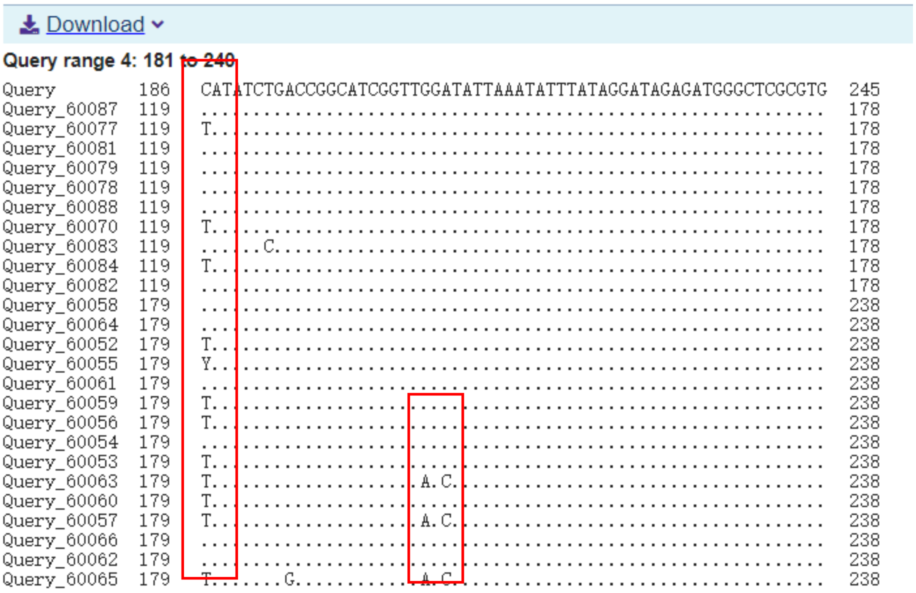

Figure S3 Differences with a clear ratio/ frequency

microorganisms

Sequences used for detection of 16S rRNA types - and their ratio - in transcriptomic level

Raw 150 bp paired reads from Illumina sequencing (LGC, GmbH, Germany) were first cleaned using fastp software (0.21.0, Chen et al. 2018). Reads with any Ns and reads length shorter than 25bp were removed. Then SortMeRna (version 4.2.0) (Kopylova et al. 2012) was used to sort out all rRNA reads, which were further used as the pool to fish out target sequences (Table S1).

Table S1 Sequences used as probes for 16S rRNA ratio of w15

Type	186	207-209	463-465--474-476	Gene sequences	Reverse complementary
C_GA	C	G-A		CATATCTGACCGGCATCGGTTGGAT	ATCCAACCGATGCCGGTCAGATATG
T_GA	T	G-A		TATATCTGACCGGCATCGGTTGGAT	ATCCAACCGATGCCGGTCAGATATA
T_AC	T	A-C		TATATCTGACCGGCATCGGTTAGCT	AGCTAACCGATGCCGGTCAGATATA
AAA_TTT			AAA-TTT	AAATACGTGTATTT	AAATACACGTATTT
TTC_GGG			TTC-GGG	TTCTACGTGTAGGG	CCCTACACGTAGAA

Analysis of taxonomic placement using alternative markers

Table S2 Similarity of 9 strains to w15 of 5 protein genes (amino acid)

Gene (bp)	rplC (206)	groL (546)	ftsA (459)	dnaE (1473)	gyrB (653)
BIGb0170	100	100	100	100	100
FDAARGOS 1141	99.51	98.90	99.78	99.32	99.23
NCTC 11343^T	100	98.90	100	98.74*	98.74*
NCTC 11034	100	98.90	100	99.12	98.93
FDAARGOS 1142	99.51	98.90	100	99.32	99.23
S.siyangensePDNC006	99.51	98.90	100	99.19	99.23
S.siyangense SY1^T	99.51	98.90	100	99.39	99.23
S.sp G1-14	99.51	98.90	100	99.39	99.23
S.spiritivorum NCTC 11386^T	94.66	94.24	80.65	83.12	88.79

*: dnaE and gyrB genes of NCTC 11343^T when translated into amino acid, multiple * were found in the sequences, thus the DSM 11691^T was used for phylogenetic analysis of marker genes.

Reference

Chen, S.; Zhou, Y., Chen, Y., Gu, J. fastp: an ultra-fast all-in-one FASTQ preprocessor, Bioinformatics **2018**, 34 (17) 1: i884–i890. doi:10.1093/bioinformatics/bty560

Kopylova, E.; Noe, L.; Touzet, H. "SortMeRNA: Fast and accurate filtering of ribosomal RNAs in metatranscriptomic data", Bioinformatics **2012**. doi:10.1093/bioinformatics/bts611.
